# Can Sequential Images from the Same Object Be Used for Training Machine Learning Models? A Case Study for Detecting Liver Disease by Ultrasound Radiomics

**DOI:** 10.3390/ai3030043

**Published:** 2022-09-01

**Authors:** Laith R. Sultan, Theodore W. Cary, Maryam Al-Hasani, Mrigendra B. Karmacharya, Santosh S. Venkatesh, Charles-Antoine Assenmacher, Enrico Radaelli, Chandra M. Sehgal

**Affiliations:** 1 Ultrasound Research Laboratory, Department of Radiology, University of Pennsylvania, Philadelphia, PA 19104, USA; 2 Department of Radiology, Children Hospital of Philadelphia, Philadelphia, PA 19104, USA; 3 Department of Electrical and Systems Engineering, University of Pennsylvania, Philadelphia, PA 19104, USA; 4 Department of Pathobiology, University of Pennsylvania, Philadelphia, PA 19104, USA

**Keywords:** medical imaging, quantitative ultrasound, machine learning, independent data, liver disease

## Abstract

Machine learning for medical imaging not only requires sufficient amounts of data for training and testing but also that the data be independent. It is common to see highly interdependent data whenever there are inherent correlations between observations. This is especially to be expected for sequential imaging data taken from time series. In this study, we evaluate the use of statistical measures to test the independence of sequential ultrasound image data taken from the same case. A total of 1180 B-mode liver ultrasound images with 5903 regions of interests were analyzed. The ultrasound images were taken from two liver disease groups, fibrosis and steatosis, as well as normal cases. Computer-extracted texture features were then used to train a machine learning (ML) model for computer-aided diagnosis. The experiment resulted in high two-category diagnosis using logistic regression, with AUC of 0.928 and high performance of multicategory classification, using random forest ML, with AUC of 0.917. To evaluate the image region independence for machine learning, Jenson–Shannon (JS) divergence was used. JS distributions showed that images of normal liver were independent from each other, while the images from the two disease pathologies were not independent. To guarantee the generalizability of machine learning models, and to prevent data leakage, multiple frames of image data acquired of the same object should be tested for independence before machine learning. Such tests can be applied to real-world medical image problems to determine if images from the same subject can be used for training.

## 1. Introduction

Image data availability is vital for the implementation of machine learning (ML) methods in clinical settings [1,2]. Large datasets with high-quality images are essential for training, validation, and testing of algorithms for clinical applications. Typically, ML algorithm performance for predictive tasks increases with increased training data volume [3–5]. If numerous parameters are to be studied, there is a need to train ML models commensurately on abundant data to obtain a generalizable model. However, there is limited access to medical images, and the preparation of image data is a costly and time-intensive process [6]. Most health care systems are not adequately equipped to share large numbers of medical images [3]. Medical data are often stored in silos and are not available in enterprise-wide core clinical systems. These data silos prevent relevant data from being shared even between departments within the same institution due to the interoperability issues of the current institutional enterprise systems [7]. Image data therefore cannot be accessed easily by AI algorithm development for widespread clinical practice.

In addition to the data availability issues, in ML a common assumption is that the given data points are realizations of independent random variables [8]. However, this assumption is often violated when the data points are highly interdependent (e.g., when the data exhibit temporal or spatial correlations) [9]. Similar scenarios are typical situations in visual recognition and computational biology [10]. Dependent data arise whenever there are inherent correlations in between observations. This is to be expected for time series of imaging data, where we would intuitively expect that instances with similar time stamps have stronger dependencies than ones that are far away in time.

A common approach to bypass the problem of limited data is to use multiple images from the same subject as separate training instances for ML [11–14]. However, this approach raises the question of whether the data are independent. Depending on the independence assumptions of the learning algorithms, the performance of the resulting models trained and tested on the same patient(s) and same body region(s) might be inflated, and the models might not be generalizable to future images. In this situation, the predictive model developed using conventional ML algorithms could be biased, inaccurate, and tend to produce unsatisfactory classifiers. A common example where ML algorithms are well known to exhibit variations in prediction accuracy is when ML is provided with imbalanced training sets due to the imbalanced ratio of pathological and normal cases typically seen in medical imaging [15]. Previous studies reported that a close-to-balanced training is required for best model performance, while data imbalance can have negative influence on the model performance.

In this study, we propose an approach to test the data independence for building a reliable diagnostic ML model using a liver disease data images set. For this purpose, we examined the independence of sequential ultrasound image frames acquired from the same cases of liver disease. We algorithmically extracted numerical liver texture features from the ultrasound images for machine learning. All these computer-generated features were used to train models. The independence between image region grayscale distributions were quantified by Jensen–Shannon (JS) divergence, a bounded symmetrization of the unbounded Kullback–Leibler (KL) divergence [16–19]. JS divergence was measured for B-mode ultrasound images acquired from images of three pathologies: normal cases, and then two groups of liver disease, namely steatosis (fatty liver) and fibrosis.

## 2. Methods

### 2.1. Image Acquisition and Computerized Analysis

1180 B-mode ultrasound images acquired in vivo from rat livers were used for analysis. The images were taken from 3 different rat groups as follows: 450 images from fibrosis cases (rats *n* = 6), 450 images from steatosis cases (rats *n* = 4), and 280 from normal (rats *n* = 4). Four video clips of B-mode images were acquired from each rat in standard transverse and sagittal imaging planes of the right and left lobes of the liver. Each clip consisted of average of 25–35 images. Imaging presets (gain = 18 dB, high sensitivity, 100% power, transmit frequency 21 MHz, and high line density) and time compensation gain were optimized and standardized.

Five to six identical rectangular regions of interest (ROI) were placed manually on each image to ensure comprehensive inclusion of multiple representative parts of the liver parenchyma and exclusion of imaging artifacts such as acoustic shadowing, enhancement, or reverberation. A total of 5903 regions of interest were placed on the images.

A number of texture features were extracted from the ROIs, which include:

1. *First order histogram features*: including echo intensity, heterogeneity (regional variance between ROIs, internal heterogeneity (local variance within ROIs) [20]. Echo intensity and heterogeneity represent the mean and standard deviation of intensity within an ROI. Heterogeneity is the standard deviation of the echo intensity between the ROIs in all the planes measured throughout the liver.
2. *Run length features* include gray-level nonuniformity (GLNU) and run length nonuniformity (RLNU). These features represent the length of the run, usually the number of pixels for the horizontal or vertical scan direction, or the number of pixels multiplied by a diagonal direction [21].
3. *Entropy:* a gray level connectivity texture feature was also studied [21].

All image analysis was performed using a custom application written in the IDL (Inter-active Data Language) programming language (version 8.5; Harris Geospatial, Broomfield, CO, USA) [21].

### 2.2. Feature Statistics and Machine Learning Diagnostic Models

The mean and standard error for the ultrasound texture features of the three different groups were compared by two-tailed paired Student’s *t*-tests. *p* < 0.05 was considered significant. One-way analysis of variance (ANOVA) was used to compare the difference between the three study arms. Statistical analysis was performed using MedCalc (version 19.0.5, MedCalc Software Ltd., Ostend, Belgium).

Two classifiers were used for machine learning analysis. Random Forest [22] was used for multicategory classification, while logistic regression is used for the two groups’ separation. Leave-one-out cross-validation approach (round-robin) was used for training and testing the data with both classifiers. Training and testing of data were performed using Weka software (version 3.8.5, University of Waikato, Hamilton, New Zealand) [23].

### 2.3. Intra- and Inter-Case Divergence Analysis

Jenson–Shannon (JS) divergence [16–19] was used to quantify the difference in grayscale distribution between two regions, for both intracase and intercase sampling. JS divergence offers an information-theoretic set-similarity measure that works naturally for pair-wise comparisons. To evaluate intra- and inter-divergence, we compared intracase to intercase pairs, calculating JS divergence for every pair. Intracase pairs were sampled for every possible time shift, and their divergence distributions were then tested against the divergence distribution of the intercase region pairs. The goal was to find the minimum time difference between image regions of the same case such that their divergences were distributed similarly to regions sample from completely different subjects. For each test at each time shift, the null hypothesis was that the distributions were different, so we performed a t-test for significant similarity or equivalence (not the more common Student’s t-test for significant difference) [24].

For equivalence, to demonstrate a “lack of difference”: The t-test for equivalence, where δ depends on how much nonequivalence is acceptable in the research study. In our example, we were unwilling to accept more than 5% reduction in intra-divergence compared to inter-divergence, so we set δ equal to 0.05 *M*_1_ (where the 1 subscript indicates inter, and 2 subscript indicates intra):

$$t(df)=\frac{M_{1}-M_{2}-\delta }{\sqrt{\left[\frac{(n_{1}-1)\sigma_{1}^{2}-(n_{2}-1)\sigma_{2}^{2}}{n_{1}+n_{2}-2}\times \left[\frac{1}{n_{1}}\frac{1}{n_{2}}\right]\right]}}$$

The denominator is simply the standard error; *df* is degrees of freedom or *n*_1_ + *n*_2_ − 2; and the *M* is mean. This was a one-sided test in this study, because we wanted to prove that intrasampled cases do not have significantly lower divergence than intercases. It is a noninferiority test because we wanted to prove that choosing our samples from intracases performed no worse than choosing from our intercases.

## 3. Histopathologic Validation

Liver disease was confirmed by histopathological examination. The liver lobes were assessed in a blind fashion by a vet pathologist for fibrosis and lipidosis on gross pathology. Portions of liver were preserved in 10% phosphate-buffered formalin and transferred to 50% ethanol after 48 to 72 h and then embedded in paraffin and processed for histological examination with hematoxylin and eosin (H&E) and trichrome staining. Each histologic section was graded according to the METAVIR scoring system for fibrosis. Lipidosis was investigated in addition to presence of balloon cells, which is critical to finding fatty liver changes.

## 4. Results

### 4.1. The Classification Performance of Ultrasound Features

All features showed statistically significant differences between the three groups. Table 1 shows the difference in mean values of liver texture ultrasound features between the three groups

**Figure 1.**
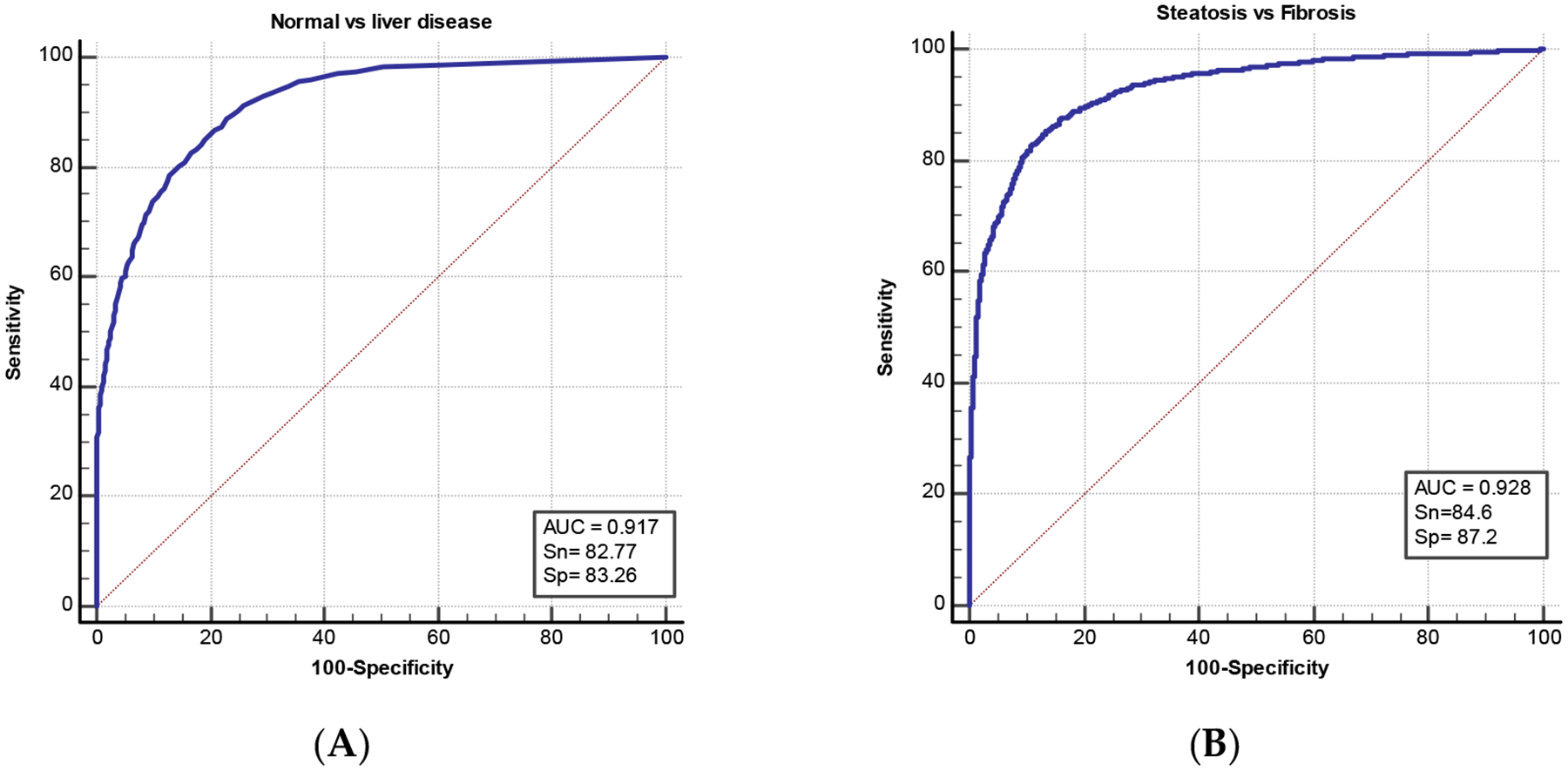
The diagnostic performance of quantitative liver texture ultrasound features using logistic regression (LR) machine learning for two-step pathology differentiation. (**A**) shows the diagnostic performance of ultrasound texture features in differentiating normal from liver disease including both fibrosis and steatosis cases, while (**B**) demonstrates the diagnostic performance of ultrasound features in differentiating steatosis from fibrosis cases. AUC refers to area under the curve, Sn: sensitivity, and Sp: specificity.

Logistic regression two-class analysis showed high performance. First, the model was able to detect the disease from normal cases with AUC 0.917 (Figure 1). Then, differentiation of the two liver disease groups, namely steatosis and fibrosis, showed a very high diagnostic performance with AUC of 0.928.

**Figure 2.**
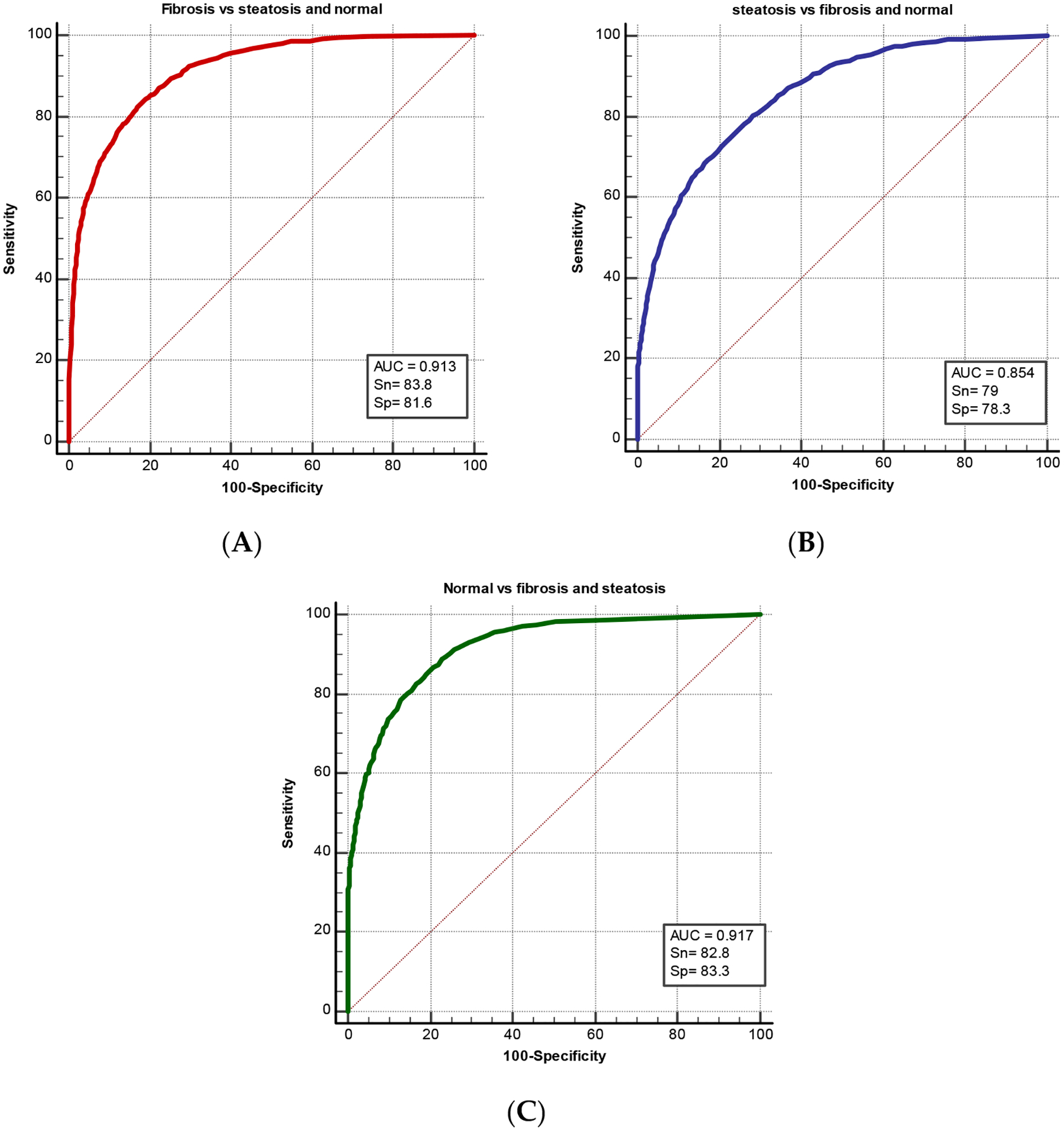
ROC curves of the diagnostic performance of quantitative liver texture ultrasound features using random forest machine learning for multicategory classification. (**A**) shows the diagnostic performance of ultrasound features in differentiating fibrosis from the two remaining groups: steatosis and normal. (**B**) demonstrates diagnostic performance in differentiating steatosis group from fibrosis and normal groups. (**C**) shows the performance of normal cases versus liver disease: fibrosis and steatosis. AUC refers to area under the curve, Sn: sensitivity, and Sp: specificity.

Random forest learning for multicategory classification also showed high performance in differentiation of the three groups (Figure 2). The model showed that the features can differentiate all three groups from each other with high diagnostic performance ranging from 0.854 to 0.917 with sensitivity up to 83.8 and specificity reaching 83.3.

### 4.2. Divergence Testing for Image Independence

**Figure 3.**
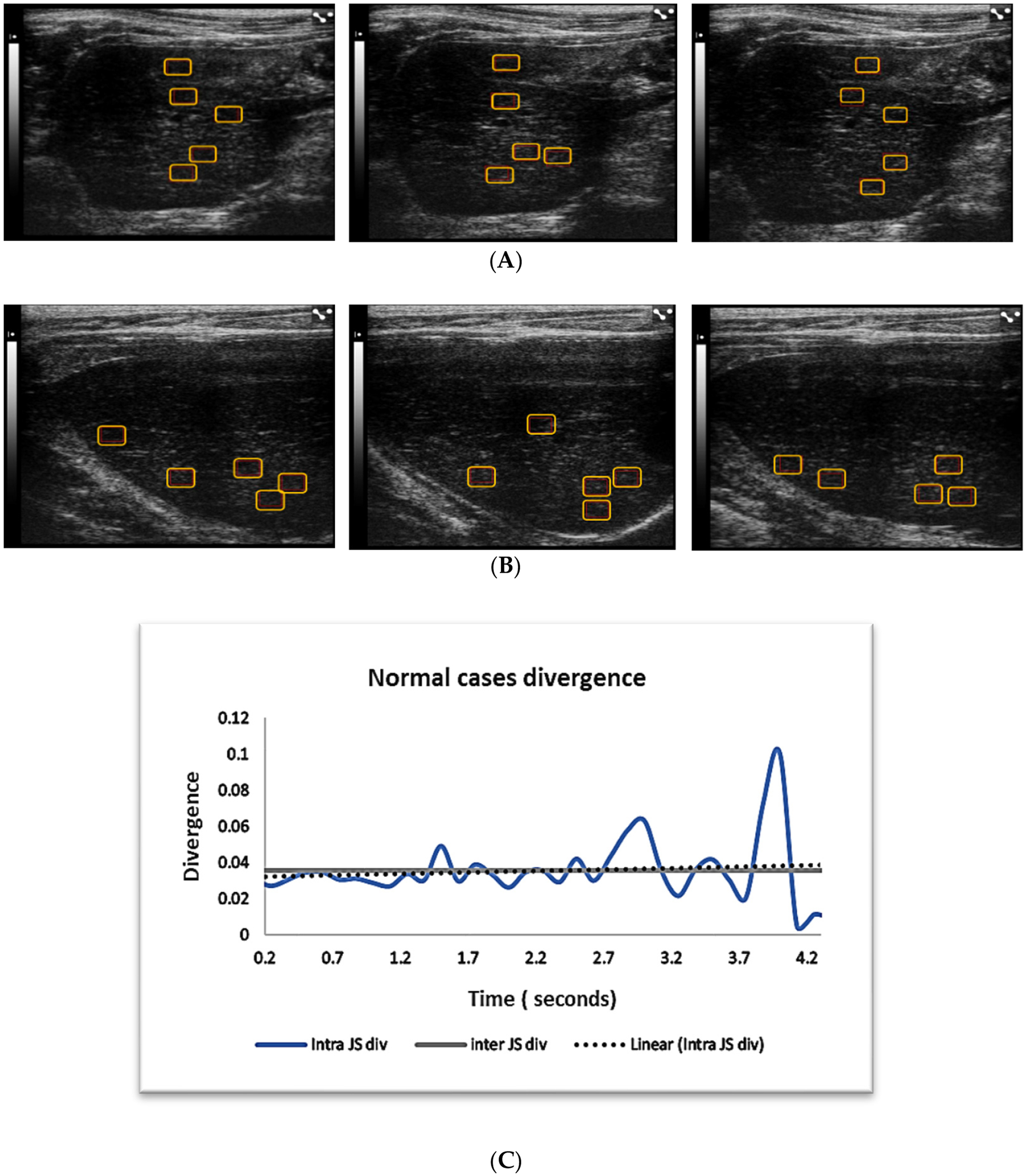
Panels (**A,B**) show examples of B-mode ultrasound liver images taken from two normal cases. Panel (**A**) shows three sequential from the same case with region of interests (ROIs) for quantitative analysis. Panel (**B**) shows three sequential images from a second normal case. Panel (**C**) shows the intra- and intercase JS divergence for cases in general. Intra- and intercase divergence for normal cases are close to each other, indicating that intrasampled cases may be just as independent inter sampling. Five to six regions of interests are placed (red rectangular boxes) on each image for quantitative analysis.

Of the three tested liver pathologies, only normal cases demonstrated that intracase region divergence is statistically close to the intersampled case divergence (Table 2, Figure 3). In Figure 3C, we can observe that the mean divergence for intrasampling was almost the same as for intersampling.

**Figure 4.**
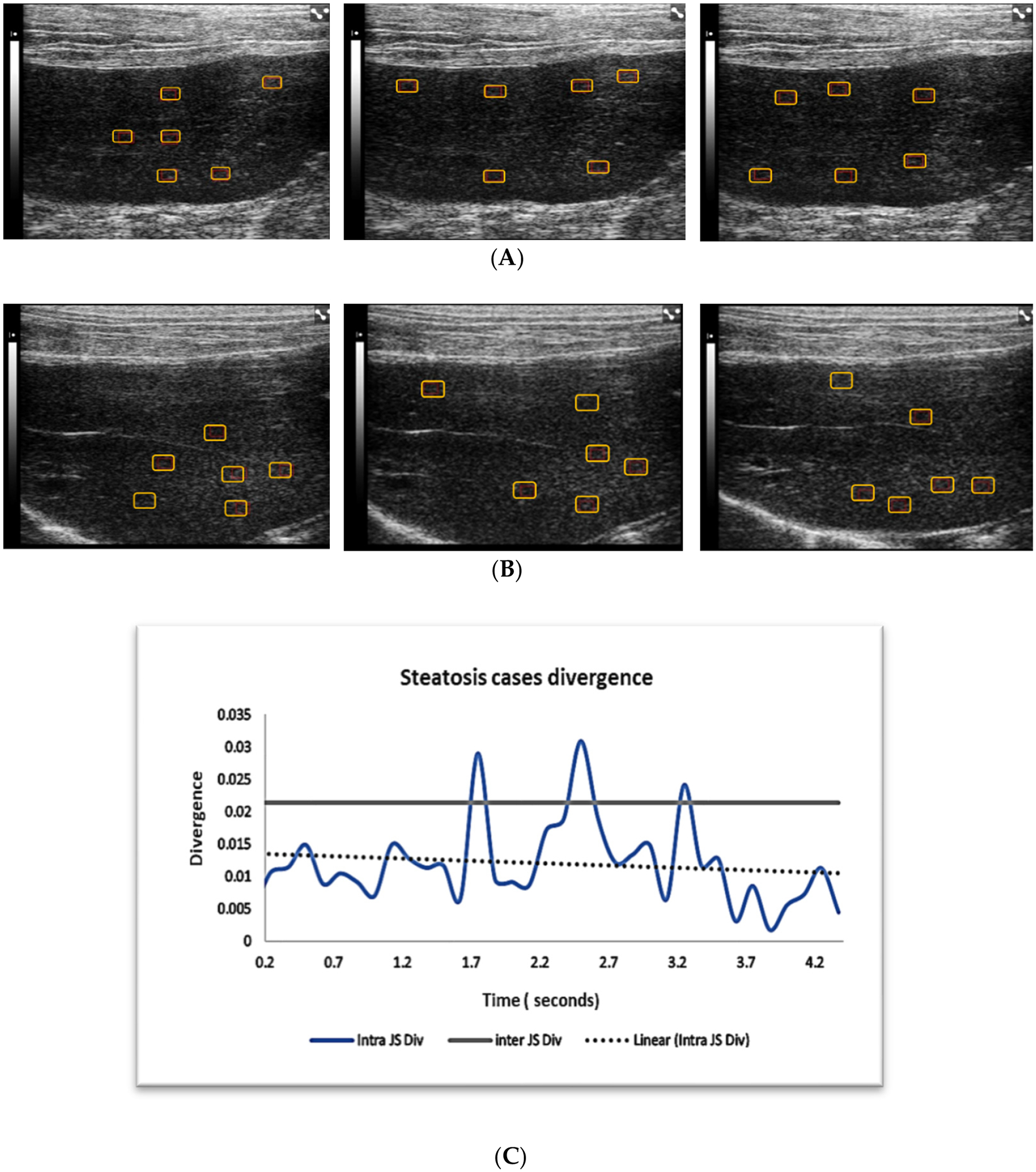
Panels (**A,B**) show examples of B-mode ultrasound liver images from two steatosis cases. Panel (**A**) shows three sequential images from the same case with region of interests (ROIs) for quantitative analysis. Panel (**B**) shows three sequential images from a second steatosis case. Panel (**C**) displays the intra- and intercase JS divergence for steatosis cases in general. Intra- and intercase divergence for steatosis cases is far apart, indicating that we cannot claim independence of intrasampling in these cases. Five to six regions of interests are placed (red rectangular boxes) on each image for quantitative analysis.

**Figure 5.**
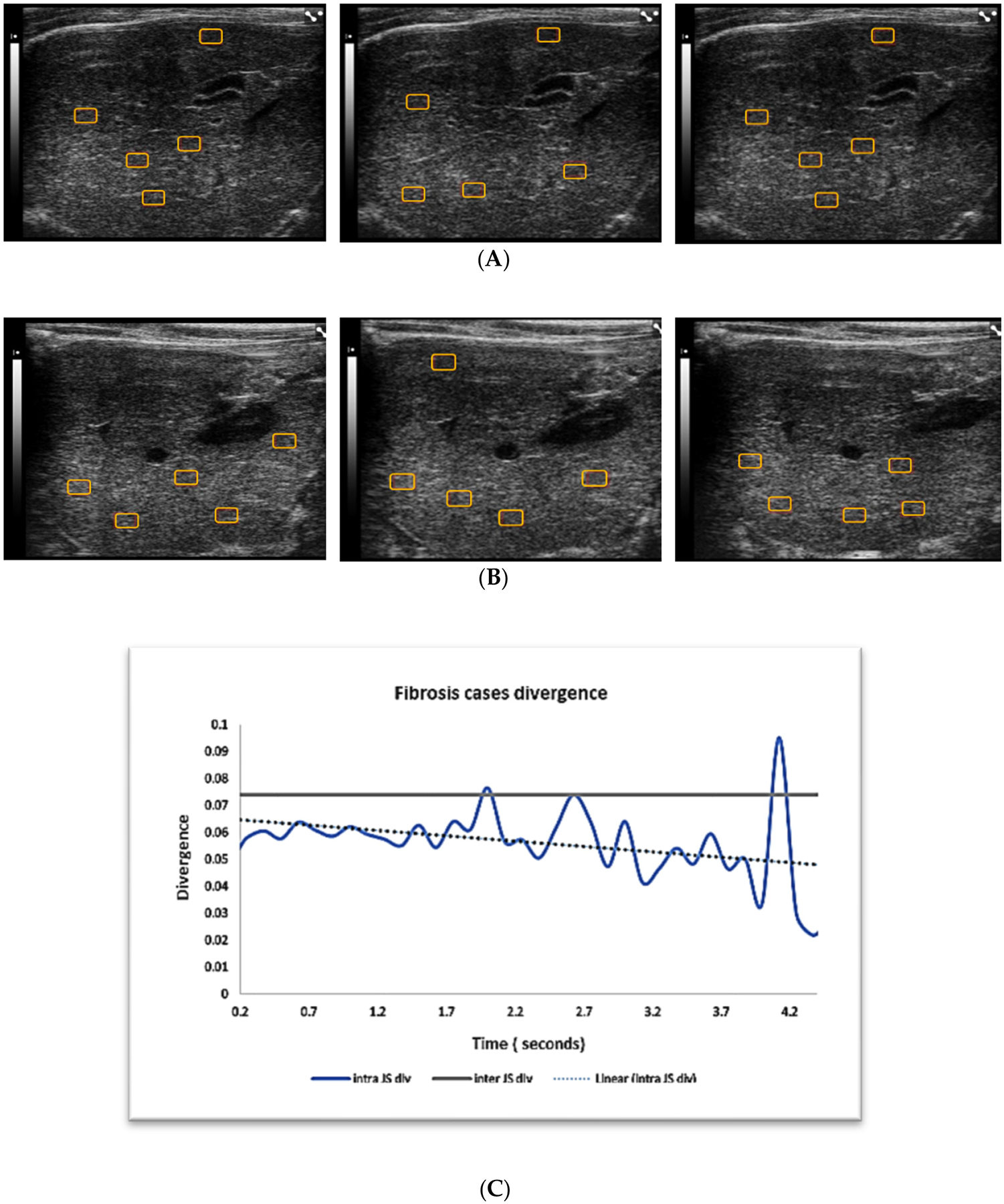
Panels (**A,B**) show examples of B-mode ultrasound liver images from two fibrosis cases. Panel (**A**) shows three sequential images from the same case with region of interests (ROIs) for quantitative analysis. Panel (**B**) shows three sequential images from another steatosis case. Panel (**C**) shows the intra- and intercase JS divergence for fibrosis cases in general. Intra- and intercase divergence for fibrosis cases is far apart, indicating that we cannot claim independence of intrasampling in these cases. Five to six regions of interests are placed (red rectangular boxes) on each image for quantitative analysis.

On the other hand, steatosis and fibrosis cases failed the similarity test. Inter-divergence was significantly higher than intra-divergence (Table 2, Figures 4 and 5): regions sampled from different cases were more different than regions sampled from the same case on different frames within a video, at different time points.

The ultrasound images examples of the three liver pathologies, in Figures 3–5, demonstrate that although it is possible to visually distinguish between the images of the three groups, the differentiation of cases within a disease group is hard on different frames of the same cases or between cases.

## 5. Discussion

For successful application of ML methods in medical imaging research and deployment of high-performance generalizable models, there is a need for a sufficient number of training samples from large images databases. We used the common training practice of adding samples by training on a large number of sequential image frames taken from the same case. The results of ML models showed high classification performance for the three studied disease groups. However, when we tested the independence of the sequential images that were taken from the same case using JS divergence, of the three tested liver pathologies, only normal cases demonstrated statistically that intracase region divergence is close to the intersampled case divergence. This means that the normal cases diverged similarly between patients and within a patient, but for fibrosis and steatosis, samples within a patient were more similar to each other than samples from different patients. Two regions sampled from the same normal case were just as different as two regions sampled from completely different cases according to the t-test for equivalence, within 5%. Therefore, for normal cases we can reject the null hypothesis that there is a significant difference between inter- and intrasampling. In general, intra-divergence for all the three groups was small, but different. Fibrosis showed the highest JS divergence in comparison to other groups. This finding is expected as fibrosis is often associated with heterogenous tissue changes between different regions of the liver in comparison to more uniform changes seen in steatosis [25,26]. Yet, inter- and intracase JS fibrosis divergence are not statistically close to each other; therefore, we cannot claim that the intracase image frames are independent enough to train on as separate cases.

The intuition that divergence should increase with time-shift between samples proved to be incorrect, because any gradual divergence trend was overpowered by the cyclical effects of breathing motion. Looking at the graphs of divergence with increasing time change, it is evident that divergence between pairs of sampled regions is periodic, with a period of 35 frames or 4.5 s. This is believed to be due to breathing. A region sampled from a later frame will be most similar (less divergent) to a region sampled earlier at the same relative point in the respiratory cycle and least similar to a region sample out of phase in the cycle. For all three tested pathologies, but especially steatosis and fibrosis, divergence change within the breathing cycle was much stronger than divergence drift over the entire time-course of the study. In theory, if this divergence oscillated perfectly with constant-period and constant-amplitude cycles, to maximize sampling differences, maximally out-of-phase time points could be chosen within or even crossing multiple periods. For instance, if the reference interdivergence was 1, and the respiratory period was 1 s (1 Hz), then the peak-to-valley maximum divergence between frames within a cycle would be at half a cycle, or 0.5 s. If the divergence difference over this half-cycle was at least equal to our reference threshold of 1, then exactly two samples that were this half-period apart, or some integer multiple of this half period, could be taken for the *entire* time of the study to ensure that they were as different from each other as two samples from entirely different cases. All of our data show that, over the time of the study, the amplitude of the divergence oscillation within a cycle increases over time: in a later breathing cycle after an earlier reference breathing cycle, a sampled region will diverge more from the earlier reference sample even when both are still in phase; and if the two samples are taken out of phase, they will also diverge much more over time. The strongest guarantee of sufficient divergence would be if the lower bound connecting the out-of-phase valleys on the increasing-oscillating curve increased by at least the reference intersampling divergence, but none of these studies showed such an effect, possibly because they were not long enough.

In this paper, we proposed the use of a statistical preliminary analysis to assess the quality of the imaging data before constructing an ML model. To our knowledge, the JS divergence test has not been evaluated to test the independence of image data. However, similar preliminary exploratory data analyses have been reported in literature for assessing the quality of data before going to ML models. One example of the exploratory analysis reported includes the use of Dynamic Time Warping (DTW) [27,28]. DTE is a measure of how similar two temporal sequences are in a time series analysis. DTW looks for the optimal alignment between the two series as opposed to looking at the Euclidean distance between two points at each time series. DTW was evaluated as a distance metric of fMRI time series with repeated measurements of an individual subject and showed that DTW analysis results in more stable connectivity patterns by reducing the within-subject variability and increasing robustness for preprocessing strategies.

One limitation of this study is that we did not test each of our measured radiomics features in this way but presented a method to perform it and tested it on a fundamental image property. Since most features depend somewhat on grayscale distribution, if ROIs between frames were not independent enough in those distributions, we did not look further. Grayscale histograms and first-order statistics are perhaps the simplest ways of characterizing image regions, so if those distributions are significantly more similar within a case than they are between cases, and more similar at certain regular time intervals within the clip, then many derived features may behave similarly. An image, though, is a spatial distribution of grayscale values, and it is very true that engineered features may only partly depend on the grayscale *value* distribution or may not depend on it at all. The methodology we presented, though, with enough samples to compare distributions (i.e., a radiomics image that maps the feature value to each pixel location) could test any quantitative feature to see if it was independent enough between frames to allow those frames to count as sufficiently independent images for that measurement. A more in-depth analysis of sampling divergence behavior over time is, however, beyond the scope of this research and would not be generalizable to other organs or modalities. The important result is that different pathologies might have different dependencies when sampling frames from video, since in this research one of three pathologies passed the independence test: sample regions between video frames of a normal case are as different from each other as samples from completely different cases. Future studies will also evaluate the effects of inter- and intrauser variability in ROI selection on the model performance. The study results are specific to ultrasound imaging, and findings could have been impacted by factors related to the choice of animal, tissue, ROI, and analysis. Future studies on a large scale are required for the proposed approach to be generalized to other imaging modalities and to be applied in human studies.

## 6. Conclusions

Whenever image frames taken from same case are used for training machine learning models, if that model assumes independence between images, an independence test should be performed. Not only might there be within-case image dependence, but that dependence also could be related to time separation of samples in a video study, so that enforcing a time interval between samples could meet the independence assumptions of the model. For our liver images, however, no such simple time interval could be found, because the periodic breathing motion was too strong an effect. However, for one of the three pathologies—the “normal” cases—image frames were as independent within a case as between cases. Such a result could be important for machine learning. In a clinical setting, it is not uncommon to acquire more disease images than normal controls, possibly creating an unbalanced imaging database. The result of this research on liver diseases suggests that it is acceptable to take many frames of the same normal case as independent training cases, demonstrating one possible application for thoroughly testing image frames for independence.

## Figures and Tables

**Table 1.** Mean ± standard errors of liver texture ultrasound features studied for the three liver pathologies. Two-sided *t*-test *p*-values are shown for each group against the other two groups. *p* < 0.05 is considered significant.

Quantitative Ultrasound Features	Steatosis	Fibrosis	Normal	*p*-Value: Fibrosis vs. Steatosis	*p*-Value: Fibrosis vs. Normal	*p*-Value: Steatosis vs. Normal
Echo intensity	34.7 ± 12.0	55.9 ± 16.3	25.4 ± 13.6	0.00	0.00	0.00
Heterogeneity	14.6 ± 3.7	20.5 ± 3.9	12.7 ± 4.7	0.00	0.00	0.0
Internal Heterogeneity	12.0 ± 1.2	16.3 ± 2.4	13.2 ± 1.8	0.00	0.00	00
GLNU	0.3 ± 0.1	0.3 ± 0.0	0.4 ± 0.2	0.00	4.37 × 10^−52^	0.00
RLNU	0.2 ± 0.0	0.2 ± 0.0	0.3 ± 0.1	0.00	1.7 × 10^−68^	2.914 × 10^−72^
Entropy	3.5 ± 0.2	4.5 ± 0.18	3.1 ± 0.5	0.00	2.4 × 10^−116^	2.041 × 10^−123^

**Table 2.** Mean and standard deviation of intra- and inter-case JS divergence values for the three pathology groups.

JS Divergence	Normal	Fibrosis	Steatosis
**Intra**	0.01 ± 0.03	0.05 ± 0.05	0.01 ± 0.05
**Inter**	0.01 ± 0.03	0.07 ± 0.08	0.03 ± 0.05

## Data Availability

The data presented in this study are available on request from the corresponding author. The data are not publicly available due to the evolving nature of the project.
